# Efficacy and safety of bipolar androgen therapy in castration-resistant prostate cancer following abiraterone or enzalutamide resistance: A systematic review

**DOI:** 10.3389/fendo.2022.1125838

**Published:** 2023-04-11

**Authors:** Xiangyun You, Shan Huang, Xin’an Wang, Cheng Yi, Niandong Gong, Junfeng Yu, Chengdang Xu, Zhendong Xiang

**Affiliations:** ^1^ Department of Urology, The People’s Hospital of China Three Gorges University, The First People’s Hospital of Yichang, Yichang, China; ^2^ Department of Urology, Tongji Hospital, School of Medicine, Tongji University, Shanghai, China

**Keywords:** bipolar androgen therapy, testosterone, castration-resistant prostate cancer, abiraterone, enzalutamide

## Abstract

Bipolar androgen therapy (BAT) is a new endocrinologic treatment for castration-resistant prostate cancer (CRPC) that can restore some patients’ sensitivity to drugs such as abiraterone (Abi) and enzalutamide (Enz). We performed a meta-analysis using STATA16. Sensitivity analyses were performed by examining the effects of individual studies using different effect models and detecting any publication bias using the Harbord test. In a total of 108 unique records, ten studies were included in the final meta-analysis. Participants who underwent BAT achieved a PSA50 response rate of 27% (95%CI [0.22,0.31], I2=17.98%), ORR of 34% (95%CI [0.24,0.43], I2=0), and incidence of AEs (grade≥3) of 14% (95%CI [0.09,0.19], I2=0). Patients who completed BAT proceeded to AR-targeted therapy (Abi or Enz) and achieved a PSA50 response rate of 57% (95% CI [0.36,0.78], I2=0). Patients with prior Enz resistance had a stronger impact on the PSA50 of AR-target therapy rechallenge. The results of this meta-analysis indicate that BAT is a safe and effective treatment for patients who have progressed after Abi or Enz. BAT can trigger the resensitization of patients with CRPC to subsequent endocrine therapy and improve the overall survival of patients and their quality of life.

## 1 Introduction

Prostate cancer (PCa) is one of the most common malignant tumors in men. With the continuous improvement in living standards and the aggravation of population aging, its incidence ranks second globally (1). Mortality ranks first in men, and fatality is lower than that in lung and colorectal cancers (2). In Asia, the incidence of prostate cancer is increasing every year (3). Some patients are in the middle and advanced stages of the disease when they are first diagnosed. As prostate cancer is an androgen-dependent tumor, androgen deprivation therapy (ADT) and inhibition of the androgen receptor signaling pathway are the main endocrinologic treatments for patients with metastatic prostate cancer (4). However, almost all patients eventually develop castration-resistant prostate cancer (CRPC) 14-30 months after ADT (5). Enzalutamide (Enz), abiraterone (Abi), and other new-generation anti-androgen drugs are more effective. However, most patients will still become resistant to new-generation anti-androgen drugs in the short term. For patients in this period, chemotherapy (such as docetaxel), molecular targeted drugs (such as sunitinib), and DNA damage repair-related poly ADP ribose polymerase inhibitors (such as olaparib) can only delay disease progression to a certain extent. There are currently no breakthrough treatments for CRPC (6, 7).

Bipolar androgen therapy (BAT) is a new treatment for patients with CRPC proposed by the Johns Hopkins University School of Medicine in recent years. The regulation of testosterone (T) between castration and supraphysiological levels suppresses cancer cell growth, thereby reducing prostate-specific antigen (PSA) and delaying castration-resistant prostate cancer progression (8). The opinion that SPA inhibits prostate cancer growth contradicts the mainstream treatment method of ADT. However, its therapeutic effect has often been verified both *in vitro* and *in vivo* in animal experiments (9–11). Clinical investigations have shown that BAT is effective (12–14). Thus, BAT is safer and more effective than cytotoxic drugs (15). BAT can restore some patients’ sensitivity to drugs, such as Abi and Enz, showing significant advantages in treating CRPC patients (16). However, there are few BAT clinical trials at present, and most are phase I and phase II clinical trials and a few case reports. There are no large phase III prospective randomized controlled trials on BAT. However, the mechanism of BAT has not yet been fully elucidated. The effectiveness and safety of BAT in clinical settings need to be further verified; there is no clear international consensus or guidance on how and when to use BAT.

Based on the above problems, we systematically analyzed the efficacy and safety of BAT in treating Abi- or Enz-resistant CRPC using a meta-analysis. We explored the best combination or sequence of BAT and current mainstream CRPC treatment drugs to provide reference and guidance for better clinical applications of BAT in the future.

## 2 Material and methods

### 2.1 Inclusion and exclusion criteria

Inclusion criteria: ① patients diagnosed with CRPC; ②Patients who failed treatment with Abi or Enz; ③ Used BAT before; ④ reported outcome indicators, such as efficacy indicators: PSA50, PSA50 response, ORR, etc. Safety indicators: AEs. Exclusion criteria: ① Documents with repeated reports; ② documents for which the full text could not be obtained or data could not be extracted.

### 2.2 Search strategy

We searched the PubMed, Embase, and Cochrane Library databases for clinical trials on BAT. The search time limit was from the establishment of the database to November 2022. In addition, the references of the included literature were traced, and the relevant literature was supplemented. The following terms were searched: “Bipolar Androgen Therapy” OR “BAT” OR “Supraphysiological Testosterone” AND “Prostate Cancer”.

### 2.3 Literature screening and data extraction

When screening the literature, the title and abstract were first read, and after excluding irrelevant literature, the full text was further read to determine whether it was finally included. The content of data extraction mainly includes ① basic information of included studies, including research title, first author, and publication time; ② baseline characteristics of research subjects, including the number of samples in each group, age of patients, etc.; ③ specific details of intervention measures, follow-up time, etc.; ④ key elements of the risk of bias assessment; ⑤ outcome indicators of concern and outcome measurement data.

### 2.4 Statistical analysis

Two researchers counted the number of participants in each study and calculated the ratio of PSA50, ORR, AEs(grade ≥3) after BAT, and PSA50 response to Abi or Enz after BAT in each study. Stata16 was used for the statistical analysis. The heterogeneity among the relevant data included in the research results was analyzed using the χ^2^ test (the test level was a=0.1), and the heterogeneity was quantitatively judged in combination with *I* ^2^. If there was no statistical heterogeneity among the research results, the fixed-effect model was used for meta-analysis, and if there was statistical heterogeneity among the research results, the random-effect model was used for meta-analysis.

## 3 Results

### 3.1 Literature screening process and results

**Figure 1:**
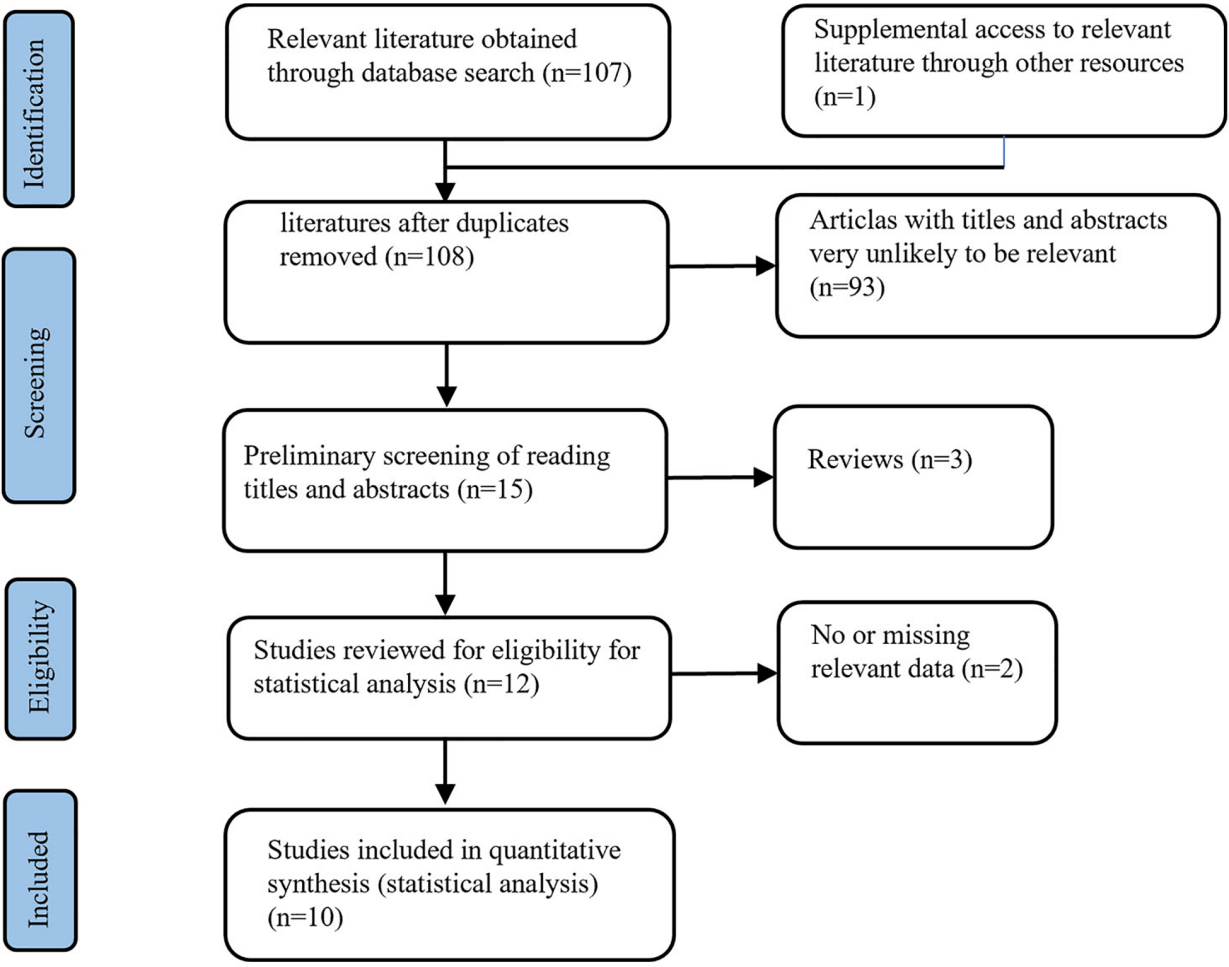
Flow chart for literature screening.

A total of 108 BAT-related studies were obtained through the primary screening. After the primary screening of titles and abstracts and rescreening of the full text, ten studies on BAT (**Figure 1**) that met the criteria were finally included in the analysis. A total of 353 patients with CRPC participated in BAT treatment.

### 3.2 Basic characteristics and results summary of the included studies

The characteristics of the included studies of BAT are summarized here (**Table 1**), such as the type of study, sample size enrolled, time of BAT treatment, and disease stage of the enrolled patients; the baseline characteristics of the included patients (**Table 2**), such as baseline PSA level, bone metastasis, ECOG score, and Gleason score at the time of pathological diagnosis of the patients enrolled in each study.

**Table 1:** Characteristics of included studies.

reference	Michael T Schweizer (13)	Benjamin A Teply (12)	Payel Chatterjee (17)	Marcus Moses (18)	Laura A Sena (17)	Samuel R Denmeade (19)	Mark C Markowski 1 (18)	Mark C Markowski 2 (18)	Senji Hoshi (20)	Liu SJ (21)	Mark C Markowski (22)
year	2015	2018	2019	2020	2021	2021	2021	2021	2022	2022	2022
journal	Sci Transl Med	Lancet Oncol	J Clin Invest	Oncotarget	Eur J Cancer	J Clin Oncol	Eur Urol	Eur Urol	Clin Case Rep	BDXXBYXB	Eur Urol Open Sci
Number of patients	16	30	62	33	29	94	29	30	4	4	22
Study type	Pilot	Prospective	Prospective	Retrospective	Prospective	Prospective	Prospective	Prospective	Case report	Case report	Prospective
BAT time, median(range)	NR	6(1-26) cycle	NR	5(2-26) cycle	NR	NR	5(1-25) cycle	NR	NR	NR	NR
PSA assessment interval	Every 2 wk	Every 4 wk	NR	NR	Every 4 wk	Every 4 wk	Every 4 wk	Every 4 wk	NR	Every 1 wk	NR
Bone/CT scans	NR	Every 12 wk	NR	NR	NR	Every 12 wk	Every 12 wk	Every 12 wk	NR	NR	NR
Type of patients	Asymptomatic mCRPC	Asymptomatic or minimally symptomatic	NR	Asymptomatic mCRPC	Asymptomatic nmCRPC or mCRPC	Asymptomatic mCRPC	Asymptomatic mCRPC	Asymptomatic mCRPC	Asymptomatic mCRPC	Asymptomatic mCRPC	Asymptomatic mCRPC
		mCRPC									

BAT, bipolar androgen therapy; CT, computed tomography; mCRPC, metastatic castration-resistant prostate cancer; NR, not reported; PSA, prostate-specific antigen; T, testosterone; Mark C Markowski 1: post Abi; Mark C MarkowskI 2: post Enz.

**Table 2:** Baseline characteristics for patients of included studies.

reference	Michael T Schweizer	Benjamin A Teply	Payel Chatterjee	Marcus Moses (18)	Laura A Sena	Samuel R Denmeade	Mark C Markowski1	Mark C Markowski2	Senji Hoshi (20)	Liu SJ	Mark C Markowski
Median age, years (range)	71 (56-87)	74 (50-89)	NR	73 (60−88)	69 (41-84)	71 (45-87)	71 (49–85)	74 (50–89)	62.5 (59-71)	74 (68-82)	NR
Median PSA, ng/ml (range)	20 (1.4-90)	39.8 (2.4-245.3)	NR	29.3 (0.04-845)	9.5 (0.5-81.1)	44.3 (1.1-323.3)	27.7 (2.8-367.9)	39.8 (3.4-245.3)	33.63 (1.4-197)	18.655 (9.939-36)	NR
Gleason score≥8,n (%)	5 (31.2)	20 (67)	NR	15 (46)	NR	60 (63.8)	16 (55)	20 (67)	3 (75)	2 (50)	NR
Bone metastasis, n (%)	3 (18.8)	21 (70)	NR	20 (61)	15 (52)	NR	NR	NR	2 (50)	3 (75)	NR
ECOG 0 (%)	15 (93.8)	22 (73)	NR	24 (73)	29 (100)	53 (56.4)	NR	NR	NR	NR	NR
Previous therapy Bicalutamide, n (%)	11 (68.8)	0	NR	0	7 (24)	NR	NR	NR	NR	4 (100)	NR
Previous therapy Abiraterone, n (%)	1 (6.3)	30 (100)	NR	16 (48)	NR	NR	NR	NR	1 (25)	1 (25)	NR
Previous therapy Enzalutamide, n (%)	3 (18.8)	13 (43.3)	NR	7 (21)	NR	NR	NR	NR	3 (75)	1 (25)	NR
Previous therapy Docetaxel, n (%)	0	0	NR	NR	6 (21)	13 (13.8)	NR	NR	0	0	NR

BAT, bipolar androgen therapy; ECOG, Eastern Cooperative Oncology Group Performance Status; GS, Gleason score; PSA, prostate-specific antigen.

### 3.3 PSA50, ORR and PSA50 response after BAT

#### 3.3.1 Results of PSA50

**Figure 2:**
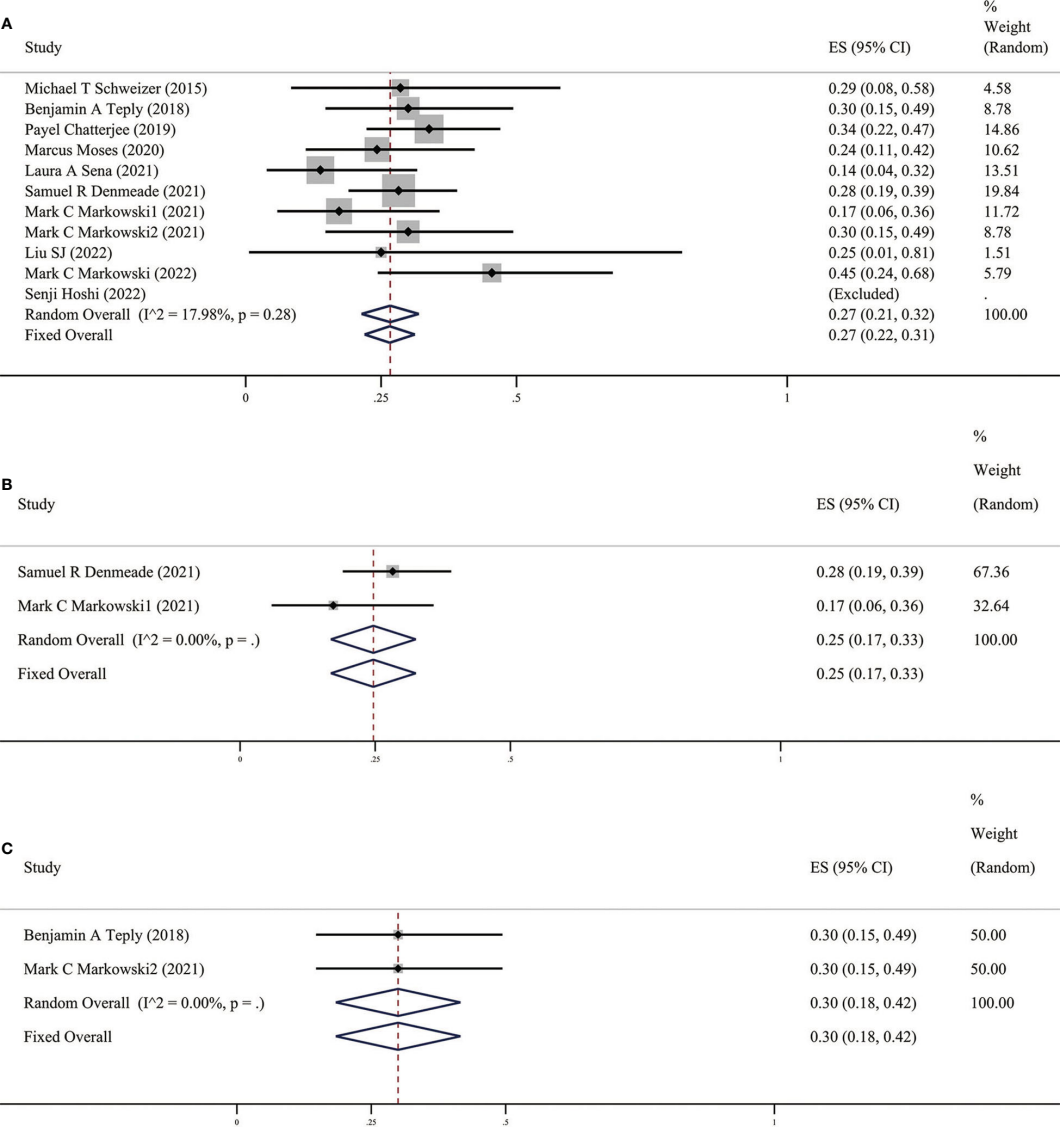
**(A)** Prevalence of PSA50 in all patients. **(B)** Prevalence of PSA50 in patients with previous Abi. **(C)** Prevalence of PSA50 in patients with previous Enz. CI, Confidence interval.

Among the ten included studies, we found no significant difference in the heterogeneity of the data between these studies (*p* > 0.05, *I* ^2^ < 90%). Therefore, the fixed-effect model was more appropriate than the random-effect model. PSA50 is defined as the PSA level of the patient after receiving BAT compared with the baseline, and the PSA level of the participant dropped by ≥50%, which is key data for evaluating the effect of BAT treatment. This was due to the heterogeneity of the enrolled patients and the unequal sample size. In these ten studies, PSA50 ranged from 14% in the study by Laura A. Sena et al (17)to 100% in the report by Senji Hoshi et al (18). Shown in **Figure 2**, a total of 353 patients were included, and the overall PSA50 response rate was 27% (95%CI [0.22,0.31], *I* ^2 =^ 17.98%). This means that nearly one-third of the patients were effectively treated with BAT, which also shows that BAT has a certain effect in clinical research.

#### 3.3.2 Results of ORR

**Figure 3:**
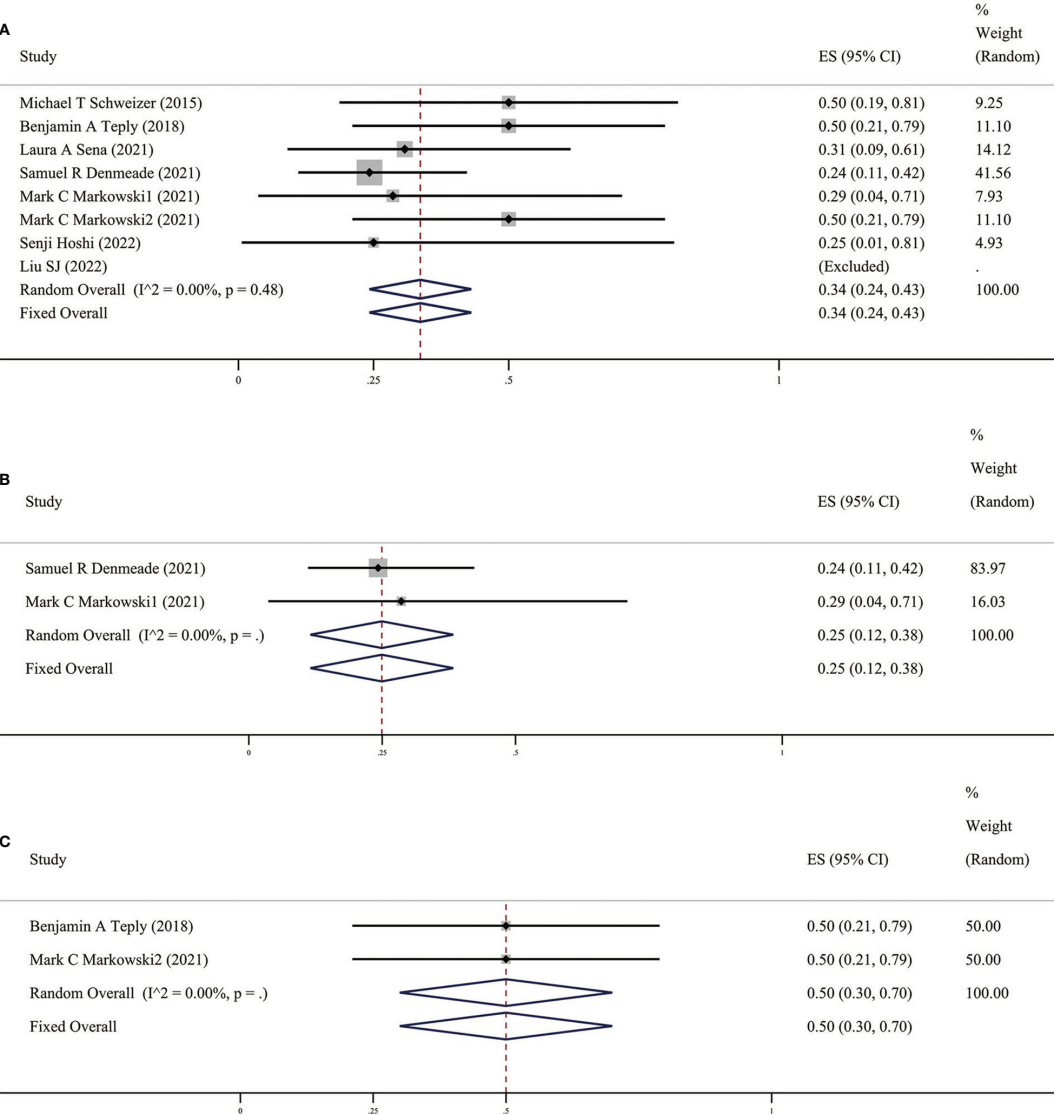
**(A)** Prevalence of ORR in all patients. **(B)** Prevalence of ORR in patients with previous Abi. **(C)** Prevalence of ORR in patients with previous Enz. CI, Confidence interval.

In clinical practice, PSA is an important indicator for prostate cancer treatment and follow-up, which doctors and patients pay great attention to. An increase in this value often indicates disease progression. The value of PSA did not increase significantly when distant metastases were detected by radiology; therefore, imaging progress and response are also key indicators for evaluating the efficacy of prostate cancer drug therapy (19). In these ten studies, patients’ objective tumor response rate (ORR) was assessed according to the Response Evaluation Criteria in Solid Tumors (RECIST), mainly through imaging examinations. ORR specifically includes partial response (PR) and complete response (CR). We found that the results of the meta-analysis in these ten studies (as shown in **Figure 3**) suggest that the overall ORR after BAT was 34% (95%CI [0.24,0.43], *I* ^2 =^ 0%). BAT has a certain effect on PSA relief and is also effective in ORR defined by imaging, which further proves the efficacy of BAT.

#### 3.3.3 Results of PSA50 response

**Figure 4:**
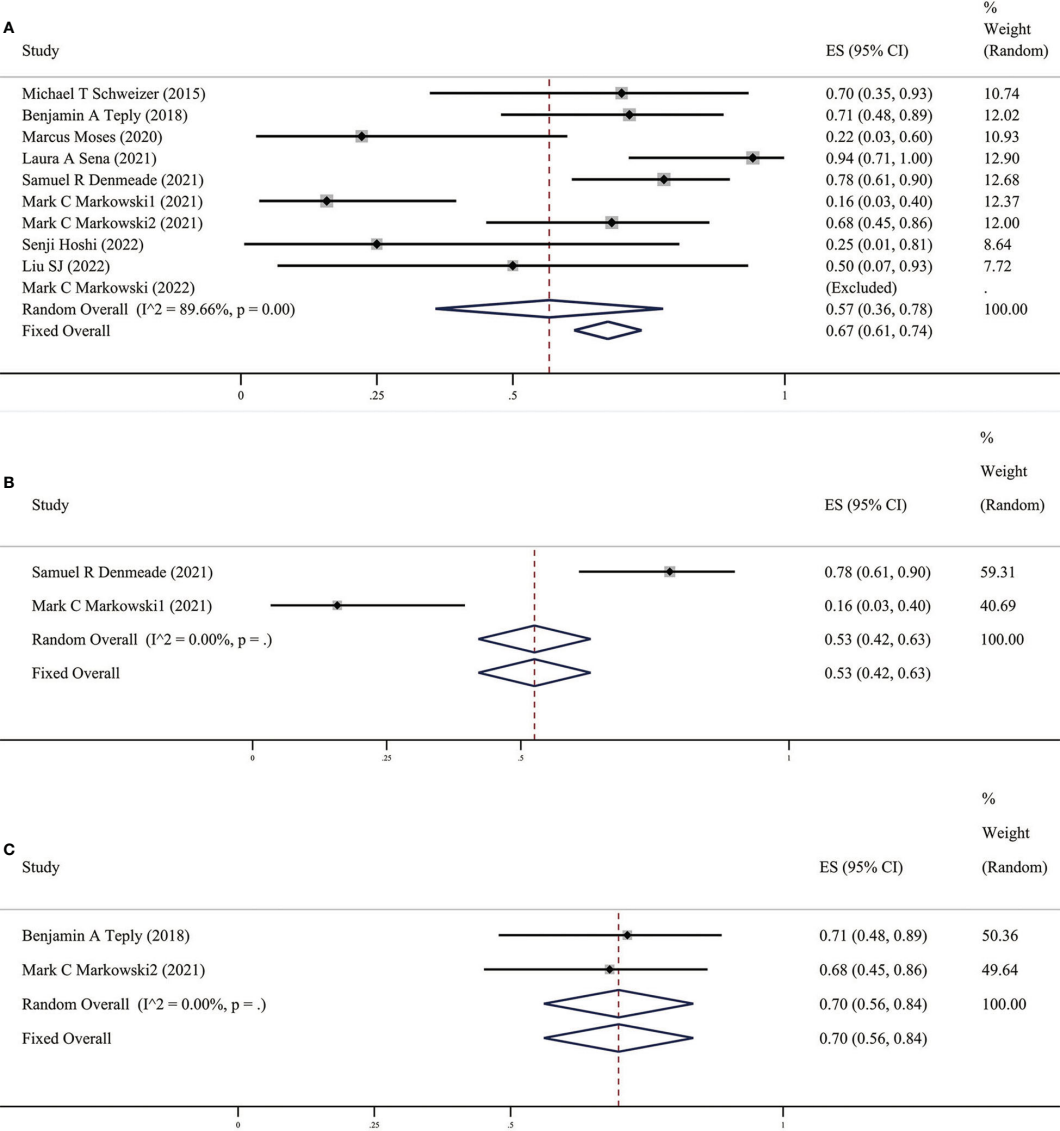
**(A)** Prevalence of PSA50 response in all patients. **(B)** Prevalence of PSA50 response in patients with previous Abi. **(C)** Prevalence of PSA50 response in patients with previous Enz. CI, Confidence interval.

As early as 2015, Schweizer et al (13) discovered and reported that 7 out of 10 CRPC patients who were originally resistant to Abi or Enz after completing the BAT cycle could respond to the new anti-Abi or Enz again. Sensitivity to androgen drug treatment: The PSA50 response rate of the ten studies included in this study (as shown in **Figure 4**) was 57% (95% CI [0.36,0.78], *I^2 =^* 0%). In other words, two-thirds of patients with CRPC who received BAT could become sensitive to antiandrogen drugs. The fact that BAT reverses the resistance of CRPC patients to Abi or Enz has undoubtedly brought new hope to patients with Abi- or Enz-resistant advanced CRPC.

### 3.4 Safety of BAT

**Figure 5:**
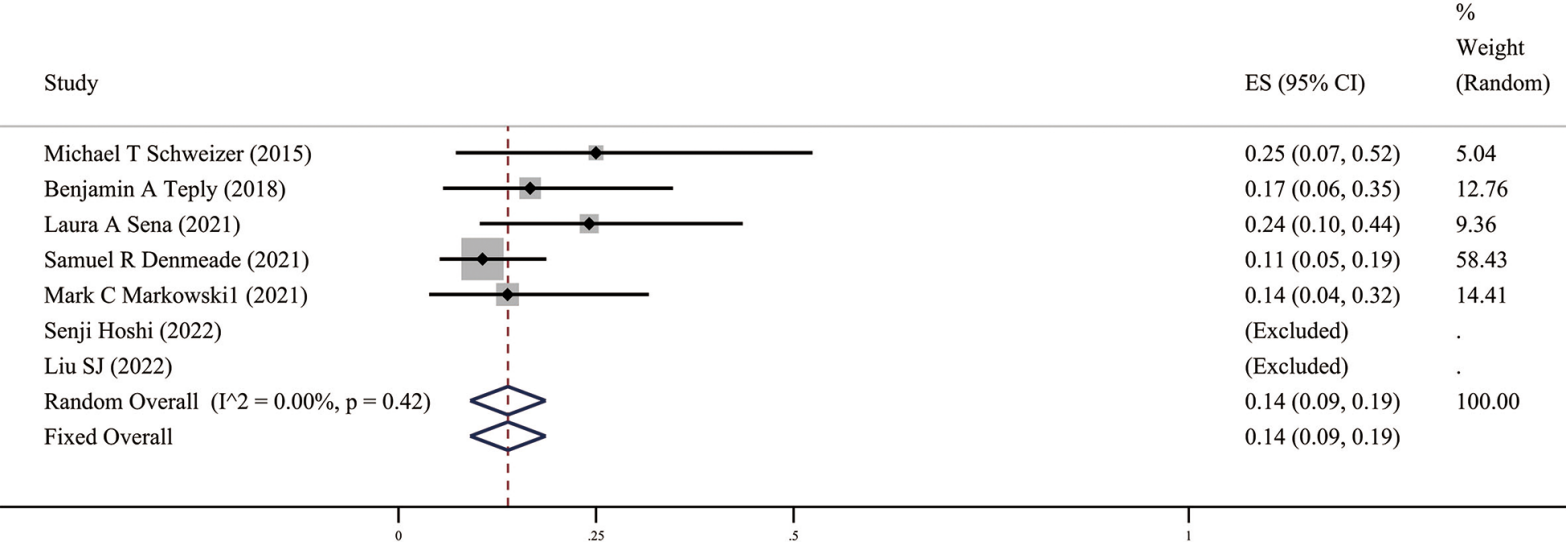
Prevalence of AEs (Grade ≥3) in patients. CI, Confidence interval.

Since androgens also have a certain effect on promoting cell growth, the issue we are most concerned about in BAT is safety, mainly including the impact of androgens, such as hot flashes, breast tenderness, breast dysplasia, and the risk of disease progression after BAT. The related AEs of the ten studies included in this study are summarized in **Table 3**, and the meta-analysis results (**Figure 5**) show that the incidence of AEs (grade ≥3) was 14% (95%CI [0.09,0.19], *I2 =* 0%).

**Table 3:** AEs (Grade ≥3) adverse events in included studies.

study	Michael T Schweizer	Benjamin A Teply	Payel Chatterjee	Marcus Moses	Laura A Sena	Samuel R Denmeade	Mark C Markowski1	Mark C Markowski2	Senji Hoshi	Liu SJ	Mark C Markowski
No. of Patients	16	30	62	33	29	94	29	30	4	4	22
Hypertension	0	3	NR	NR	2	0	0	NR	0	0	NR
Pulmonary embolism	2	0	NR	NR	1	0	0	NR	0	0	NR
Back pain	0	0	NR	NR	0	3	0	NR	0	0	NR
Fatigue	0	0	NR	NR	0	0	0	NR	0	0	NR
Musculoskeletal pain	0	0	NR	NR	1	4	1	NR	0	0	NR
Urinary obstruction	0	1	NR	NR	1	0	0	NR	0	0	NR
Neutropenia	1	0	NR	NR	0	0	0	NR	0	0	NR
Elevated Alk Phos	0	0	NR	NR	0	0	1	NR	0	0	NR
DIC	0	0	NR	NR	0	0	1	NR	0	0	NR
Bowel obstruction	0	0	NR	NR	0	0	1	NR	0	0	NR
Gallstone	0	0	NR	NR	0	0	0	NR	0	0	NR
Sepsis	0	1	NR	NR	0	0	0	NR	0	0	NR
NSTSEMI	0	0	NR	NR	0	0	0	NR	0	0	NR
Death	1	0	NR	NR	0	0	0	NR	0	0	NR
Stroke	0	0	NR	NR	1	0	0	NR	0	0	NR
Thrombocytopenia	0	0	NR	NR	0	0	0	NR	0	0	NR
Myocardial infarction	0	0	NR	NR	1	0	0	NR	0	0	NR
Edema limbs	0	0	NR	NR	0	1	0	NR	0	0	NR
Nausea	0	0	NR	NR	0	1	0	NR	0	0	NR
Hematuria	0	0	NR	NR	0	1	0	NR	0	0	NR

NR, not reported; DIC, disseminated Intravascular Coagulation.

### 3.5 Results of survival data after the BAT

Survival data of BAT mainly include time to PSA progression (TPP), no radiographic progression-free survival (rPFS), PFS2 (progression-free survival 2), and radiographic progression-free survival (crPFS). The number of studies on data completeness varies greatly, as summarized in **Table 4**.

**Table 4:** Descriptive results of survival data.

study	TPP, median (95% CI/range), mo	rPFS, median (95% CI/range), mo	crPFS, median (95% CI/range), mo	PFS2
Michael T Schweizer	7.37 (3.17-15.13)	NR	NR	12.8 (7.3-16.3)
Benjamin A Teply	3.3 (2.7-5.5)	NR	6.5 (4.3-8.0)	
Payel Chatterjee	NR	NR	NR	
Marcus Moses	0.9 (95% CI, 0.3–1.4)	NR	NR	
Laura A Sena	not been reached	8.5 (95% CI, 6.9-15.1)	NR	
Samuel R Denmeade	2.79 (95% CI, 1.81-4.5)	5.75 (5.55−8.41)	5.62 (4.76-5.91)	28.2 (23.6-NR)
Mark C Markowski	NR	5.0 (3.3-9.3)	4.3 (3.3-5.3)	8.1 (6.1-9.5)
Senji Hoshi	NR	NR	NR	
Liu SJ	NR	NR	NR	

TPP, time to PSA progression; rPFS, radiographic progression-free survival; crPFS, clinical or radiographic progression-free survival; PFS2, progression-free survival 2; NR, not reported.

## 4 Discussion

Prostate cancer progression depends on androgens, therefore, reducing androgen levels or blocking the androgen receptor (AR) can inhibit prostate cancer (20). During ADT, prostate cancer cells can regulate AR activity through AR gene amplifications, mutations, and post-translational modifications to adapt to the chronic androgen deprivation environment. However, the castration level of androgens and overexpression of AR leads to increased sensitivity of CRPC cells to supraphysiological levels of androgens, further inhibiting DNA replications and inducing double-strand DNA breaks, inhibiting tumor cell growth, and promoting apoptosis. Based on the different responses of prostate cancer cells to different levels of androgens, BAT inhibits CRPC cells through the sudden rise and fall of androgen levels and rapid alternation and delays the disease progression in the CRPC stage. The results of our meta-analysis also confirmed the effectiveness and safety of BAT for CRPC patients who progressed after Abi or Enz, with a PSA50 rate of 27% and an ORR of 34%. BAT can also trigger the resensitization of CRPC patients to subsequent endocrine therapy; the PSA50 response rate was 67%, and the incidence of AEs (grade ≥3) after BAT treatment was 14%.

Xiong et al (8) proved the safety and effectiveness of this therapy in a previous meta-analysis, which played a guiding role in the promotion and application of BAT. However, the included clinical studies and the total number of people were not many; with the increase in BAT clinical trials, we updated the included BAT clinical studies. There have been a few case reports of BAT performed in individual clinical research centers. The results showed that the PSA50 response rate was significantly increased (67% vs. 54%), and the overall safety and effectiveness were better, further demonstrating the possibility, safety, and effectiveness of BAT in optimizing traditional endocrine therapy.

It is interesting that there are differences in BAT treatment efficacy in patients with previous Abi or Enz, therefore, we grouped the earlier BAT treatment studies with Abi or Enz treatment. We found that the PSA50 rate of patients with previous Abi was 25% (as shown in **Figure 2B**), ORR was 25% (as shown in **Figure 3B**), the PSA50 response rate was 53% (as shown in **Figure 4B**). But in the Enz group, the PSA50 rate of patients was 30% (as shown in **Figure 2C**), the ORR was 50% (as shown in **Figure 3C**), and the PSA50 response rate increased to 70% (as shown in **Figure 4C**) in patients continued to receive Enz after progression. According to the above results, re-sensitivity to novel hormonal therapy (NHA) can be achieved through BAT, thus prolonging the effectiveness of NHA. BAT resulted in a higher PSA50 and ORR in patients treated with Enz than in those treated with Abi. BAT is a better choice for patients and doctors and can be added to the NHA treatment base, in particular, BAT sequential Enz can further improve the curative effect.

In terms of safety, most of the AEs of BAT are grade 1-2, including fatigues, generalized pains, and lower extremity edema (8). Our results suggest that the incidence of AEs (grade≥3) was 14%. Compared to the incidence of AEs corresponding to Enz or Abi (21), BAT can significantly improve a patient’s quality of life. This shows that the BAT regimen can be reasonably applied in patients with advanced CRPC resistant to Abi or Enz.

Simultaneously, BAT can be used to predict patient prognosis. Denmeade et al (22) showed that a peak PSA level of less than 9 ng/ml after BAT treatment was associated with a longer duration of ADT response; PSA≥9 ng/ml (n=16) patients had a median progression-free survival of 20.6 months, while the median overall survival of patients with peak PSA <9 ng/ml was 79.6 months. In current clinical trials, it was also found that not all patients were sensitive to BAT treatment. It is important to identify suitable biomarkers to distinguish BAT responders from non-responders. With the current results, markers of response to BAT are still being explored (23), which is also the direction of our future research.

However, some studies included in this study (such as the case reports of Senji et al (18) and Liu et al (24) may have an impact on the results of this study due to the small number of cases.

## 5 Conclusions

The results of this meta-analysis indicate that BAT is a safe and effective treatment for patients who have progressed after Abi or Enz. BAT can trigger the resensitization of patients with CRPC to subsequent endocrine therapy and improve the overall survival of patients and their quality of life. However, biomarkers that can predict the efficacy of BAT still need to be identified.

## Data availability statement

The original contributions presented in the study are included in the article/**Supplementary Material.** Further inquiries can be directed to the corresponding authors.

## Author contributions

XY, SH: Protocol development, Data collection or management, Data analysis, Manuscript writing and editing. CY, XW: Manuscript writing and editing. JY, NG: Data collection and management. ZX, CX: Protocol development, Data analysis, Manuscript editing. All authors contributed to the article and approved the submitted version.
