# Supplementary material for: Efficacy and safety of bipolar androgen therapy in castration-resistant prostate cancer following abiraterone or enzalutamide resistance: A systematic review
Source: Front Endocrinol (Lausanne). 2023 Apr 11;13:1125838. doi: 10.3389/fendo.2022.1125838 (PMC10127253; doi:10.3389/fendo.2022.1125838)
Supplement: Supplementary file 1 [file DataSheet_1.docx]

Supplementary Material

Efficacy and safety of bipolar androgen therapy in castration-resistant prostate cancer following abiraterone or enzalutamide resistance: a systematic review

Xiangyun You^1†^, Shan Huang^1†^, Xin’an Wang^2^, Cheng Yi^1^, Niandong Gong^1^, Junfeng Yu^1^, Chengdang Xu^2*^, Zhendong Xiang^1*^

*** Correspondence:**Zhendong Xiang: [zhendong@ctgu.edu.cn](mailto:zhendong@ctgu.edu.cn);

Chengdang Xu: xuchengdang1990@163.com


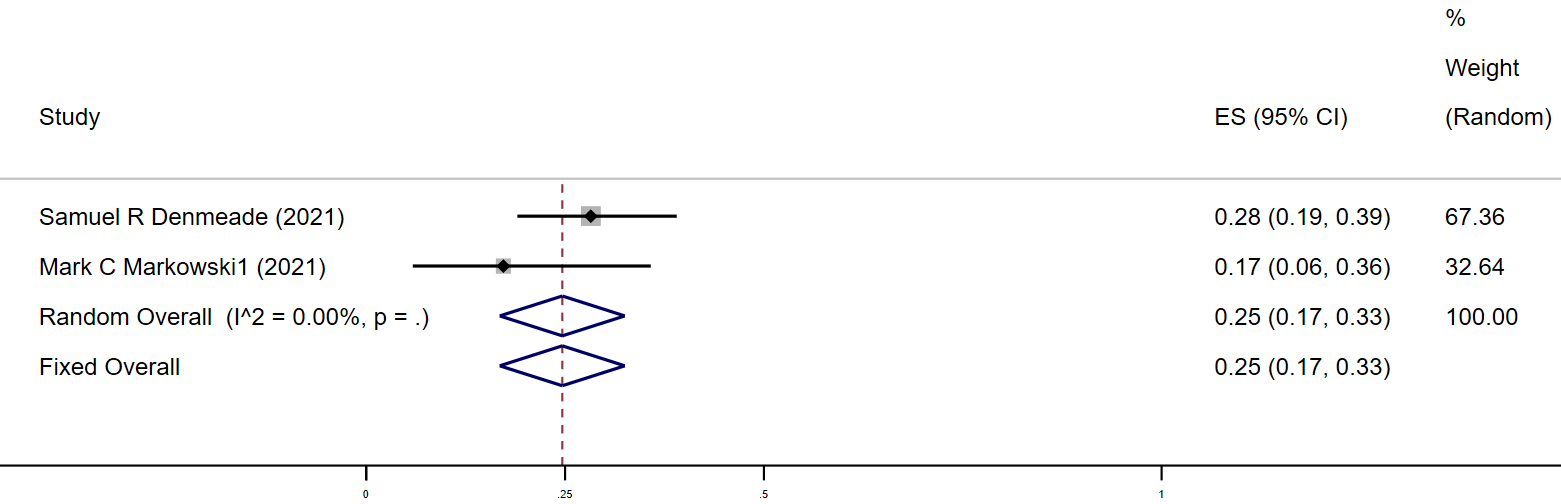


**Supplementary Figure 1** Prevalence of PSA50 in Abi patients. CI, Confidence interval.


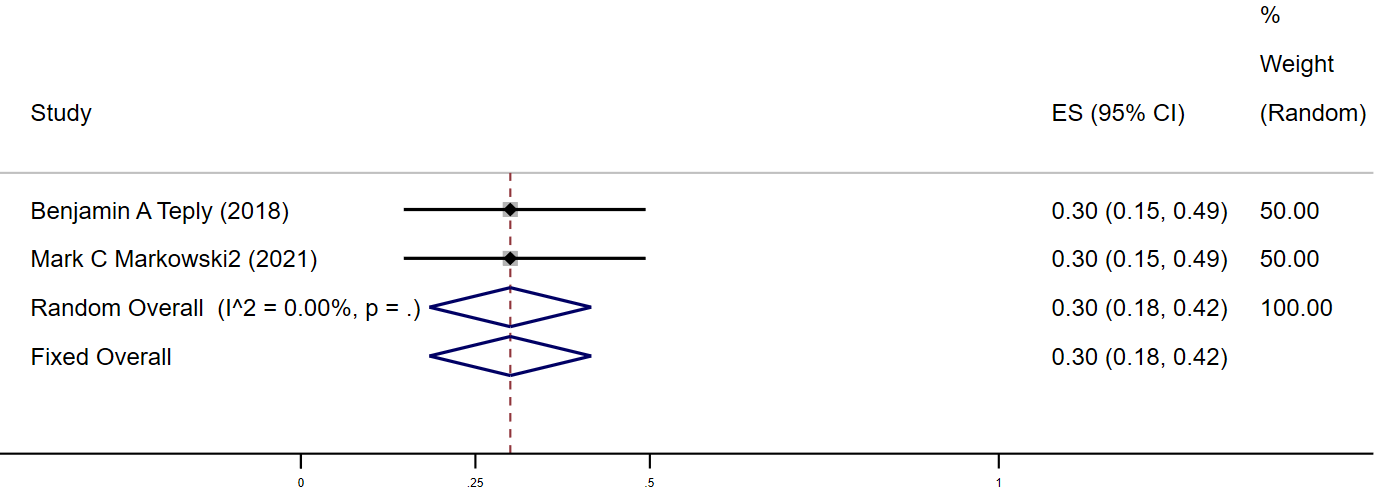


**Supplementary Figure 2** Prevalence of PSA50 in Enz patients. CI, Confidence interval.


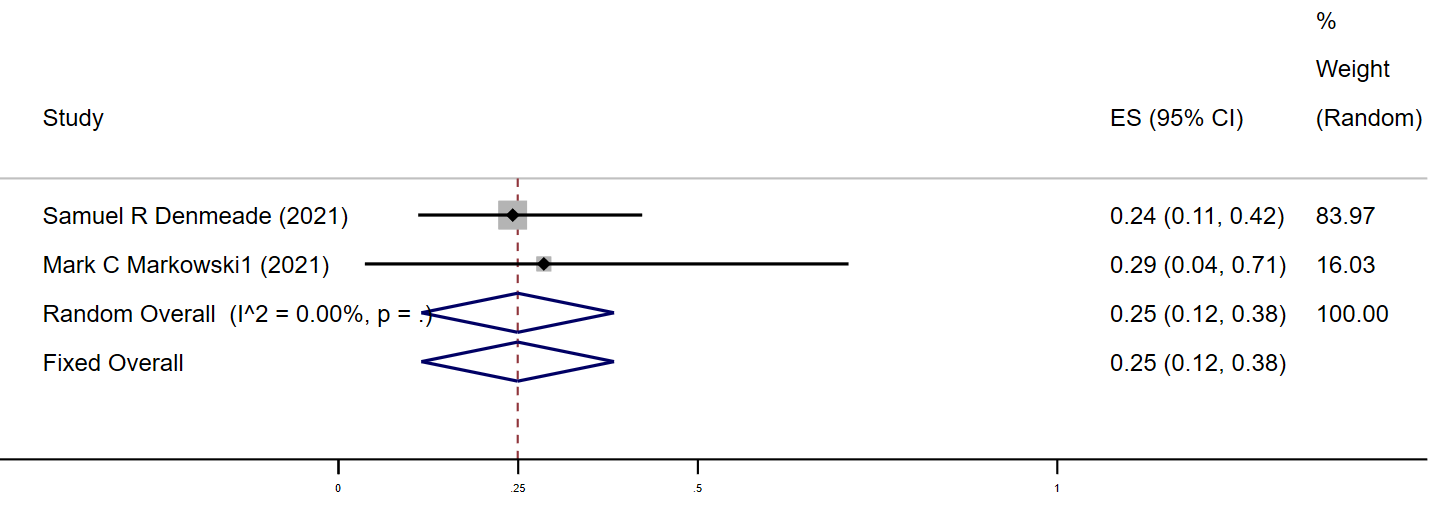


**Supplementary Figure 3** Prevalence of ORR in Abi patients. CI, Confidence interval.


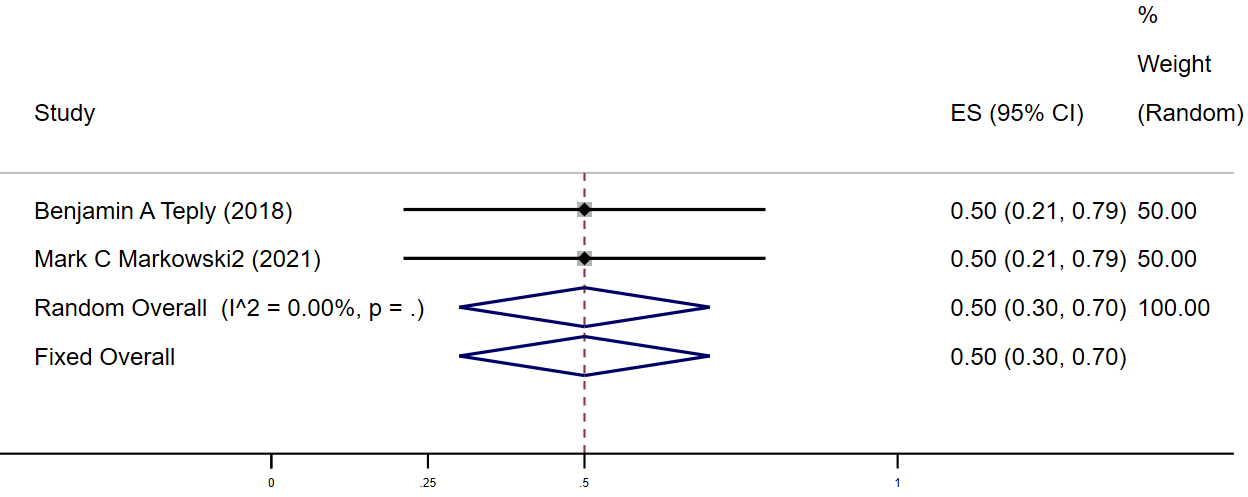


**Supplementary Figure 4** Prevalence of ORR in Enz patients. CI, Confidence interval.


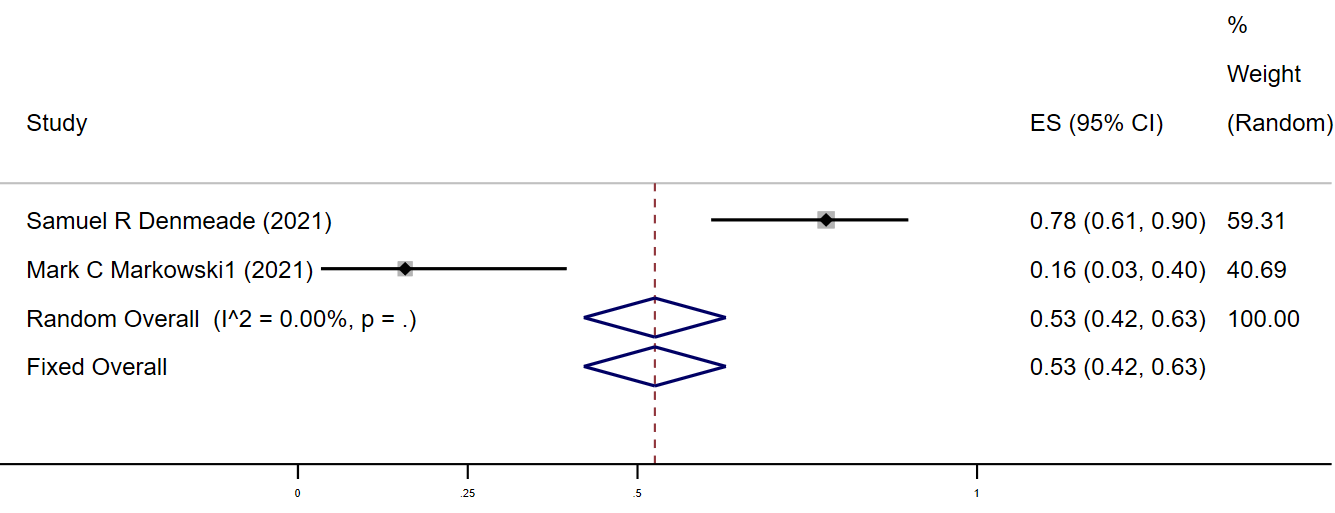


**Supplementary Figure 5** Prevalence of PSA50 response in Abi patients. CI, Confidence interval.


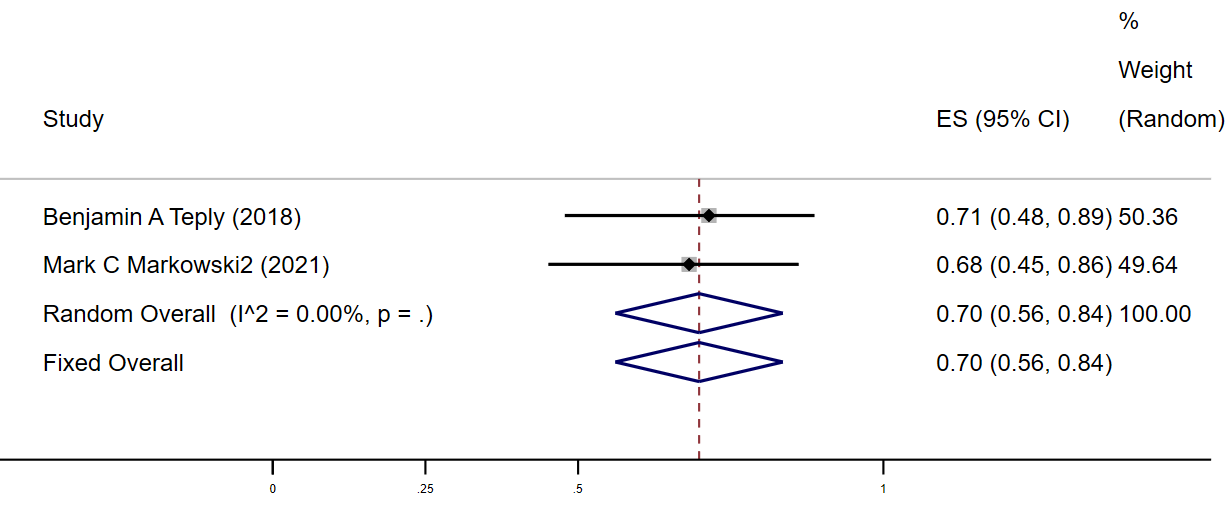


**Supplementary Figure 6** Prevalence of PSA50 response in Enz patients. CI, Confidence interval.
